# Editorial: From the mouth to the brain: exploring the link between periodontitis/peri-implantitis and neuroinflammation

**DOI:** 10.3389/fncel.2026.1944333

**Published:** 2026-08-13

**Authors:** Gestter Willian Lattari Tessarin, Dirk M. Hermann

**Affiliations:** 1 School of Dentistry, University Center in the North of São Paulo (UNORTE), São Paulo, Brazil; 2 Department of Neurology, University Hospital Essen, University of Duisburg-Essen, Essen, Germany

**Keywords:** infection, injury, mouth–brain axis, neurodegeneration, neuropsychiatric

In the central nervous system (CNS), it is essential that its various cells—including astrocytes and microglia—operate in a coordinated manner to maintain brain physiological functions (Sofroniew and Vinters, 2010; McKinney et al., 2026). When nervous tissue homeostasis is disrupted, for example by microbial infection and/or its toxins, cerebral danger responses are activated in microglial cells, leading to neuroinflammation through the production and release of cytokines and chemokines (Reis and Castro-Faria-Neto, 2022). Neuroinflammation aims at protecting the CNS under attack against injury (Reis and Castro-Faria-Neto, 2022). During neuroinflammation, astrocytes and microglial cells undergo profound structural, metabolic, and transcriptional reprogramming, which may drive them toward highly inflammatory or neurotoxic phenotypes (Han et al., 2025; García-Domínguez, 2026; Sharma et al., 2026). Although this response is initially protective, it can promote progressive synaptic dysfunction, neuronal damage, cognitive and motor impairment, when getting chronic or dysregulated. Thus, persistent neuroinflammation has been implicated in the pathogenesis and progression of several neurodegenerative disease, including Alzheimer's disease, Parkinson's disease, multiple sclerosis, and amyotrophic lateral sclerosis (Kempuraj et al., 2016; Balistreri and Monastero, 2023; Adamu et al., 2024; Cohen et al., 2024).

The mouth–brain axis has emerged as a transformative framework for understanding how oral inflammatory diseases driven by microorganisms may influence CNS homeostasis (for review, da Conceição Francisquini et al., 2025; Tessarin et al.; Xu et al., 2026). Beyond their local effects within the oral cavity, periodontitis and peri-implantitis are increasingly recognized as potential sources of systemic immune activation that may initiate and/or exacerbate inflammatory processes through multiple interconnected pathways (Bui et al., 2019; Sansores-España et al., 2021; Cafferata et al., 2025). In general terms, neuroinflammation may be initiated or sustained through dissemination of periodontal and peri-implant pathogens and their virulence factors via the blood, translocation of bacterial extracellular vesicles, persistent release of pro-inflammatory cytokines, disruption of blood–brain barrier integrity, and trafficking of peripheral immune cells. Together, these mechanisms can promote glial activation and amplify inflammatory

responses in the CNS (Hajishengallis and Chavakis, 2021; Li et al., 2022; Dumitru et al., 2026; Tessarin et al.). Although direct causal evidence in humans remains limited, a growing body of experimental and clinical evidence supports the biological plausibility of bidirectional communication between the oral cavity and the CNS (Ball and Darby, 2022; Kichenbrand et al., 2020; Koseki et al., 2020; Li et al., 2022; Takahashi et al., 2022; Cafferata et al., 2025; Kong et al., 2025; Cafferata and Schwarz, 2026). Consequently, the mouth–brain axis has evolved from a theoretical concept into a rapidly expanding field of research that integrates dentistry, neuroscience, immunology, microbiology, and medicine. Elucidating the mechanisms underlying this complex communication network may not only advance our understanding of neuroinflammatory and neurodegenerative disorders but also identify oral diseases as potentially modifiable contributors to nervous tissue health, thereby opening new opportunities for preventive strategies, biomarker discovery, and interdisciplinary therapeutic interventions.

The “*From the mouth to the brain: exploring the link between periodontitis/peri-implantitis and neuroinflammation*,” Research Topic brought together *in vivo* and *in vitro* studies, comprehensive literature reviews, and expert perspectives to explore the complex interconnections between oral dysbiosis, periodontitis, peri-implantitis, and neuroinflammation. Collectively, these contributions examined the biological pathways underlying the mouth–brain axis while also highlighting potential therapeutic strategies, including the antimicrobial properties of novel plant-derived compounds. Among the original research articles, Dai et al., using a rat model, demonstrated that the gram-negative anaerobic bacterium *Porphyromonas gingivalis*, a frequent cause of severe and aggressive periodontitis, promotes CNS inflammation by driving Th1-cell differentiation through activation of the peripheral ZAP70/NF-κB signaling pathway. Miranda et al. reported that extracts of the medicinal plants *Juglans regia* and *Pfaffia paniculata* exhibit promising antimicrobial activity against oral dysbiosis-associated microorganisms, suggesting their potential as adjunctive treatments to mitigate oral dysbiosis. Using human samples and animal models, Zhou et al. demonstrated that STAT3-mediated Th17-cell pathogenicity represents a mechanistic link between periodontitis and neuroinflammation associated with cognitive impairment. The review article carried out by Zhang et al., postulated that periodontitis may represent a modifiable risk factor for Alzheimer's disease and that periodontal interventions could contribute to disease prevention and management. In their opinion article, Tessarin et al. proposed that extracellular vesicles released by microorganisms in association with peri-implantitis may act as biological vectors capable of promoting neuroinflammation through the blood and lymphatic systems, as well as along peripheral nerve fibers.

Collectively, the studies included in this Research Topic reinforce the concept that oral dysbiosis and inflammatory diseases affecting periodontal and peri-implant tissues should no longer be regarded as conditions confined to the oral cavity. Instead, they represent biologically active processes capable of influencing distant organs through multiple interconnected mechanisms. Although each contribution addresses a distinct aspect of the mouth–brain axis, together they provide complementary evidence supporting the existence of diverse biological pathways linking the oral cavity to the nervous tissue and highlighting their potential role in the initiation and maintenance of neuroinflammation. Considering that the brain is an extraordinarily complex organ (Feher, 2012; Caradonna et al., 2024; Ali et al., 2025; Maldonado and Alsayouri, 2026), while the oral cavity is a remarkably multifaceted ecosystem (Baker et al., 2024), the biological pathways and communication axes that mediate deleterious interactions between the oral cavity and the brain remain incompletely characterized. Thorough mechanistic studies will be needed to explore the mechanistic links between periodontal and peri-implant diseases, neuroinflammation, and brain injury.

It should also be recognized that the relationship between mouth-brain is likely bidirectional. Neurodegenerative diseases may profoundly influence the oral environment by impairing oral hygiene practices, reducing salivary secretion, altering the composition of oral microbiota, and disrupting local immune homeostasis (Ide et al., 2016; Delwel et al., 2018; Wan and Fan, 2023). Collectively, these alterations may promote oral dysbiosis and periodontal and peri-implant inflammation, thereby establishing a self-perpetuating feedback loop that amplifies inflammatory responses. In this way, this interaction should also be viewed within the broader framework of a “brain–mouth–brain” axis rather than as a strictly unidirectional pathway.

We hope that this Research Topic will stimulate further interdisciplinary research and contribute to establishing the oral–CNS axis as a mature and rapidly evolving field, capable of generating innovative preventive, diagnostic, and therapeutic strategies for both oral and neurological diseases.
